# Aldosterone does not alter endothelin B receptor signaling in the inner medullary collecting duct

**DOI:** 10.14814/phy2.13167

**Published:** 2017-03-07

**Authors:** Nirupama Ramkumar, Deborah Stuart, Tianxin Yang, Donald E. Kohan

**Affiliations:** ^1^ Division of NephrologyUniversity of Utah Health Sciences CenterSalt Lake CityUtah; ^2^ Salt Lake Veterans Affairs Medical CenterSalt Lake CityUtah

**Keywords:** Aldosterone, collecting duct, endothelin, endothelin B receptor, signaling

## Abstract

Recent studies suggest that aldosterone‐mediated sulfenic acid modification of the endothelin B receptor (ETB) promotes renal injury in an ischemia/reperfusion model through reduced ETB‐stimulated nitric oxide production. Similarly, aldosterone inactivation of ETB signaling promotes pulmonary artery hypertension. Consequently, we asked whether aldosterone inhibits collecting duct ETB signaling; this could promote fluid retention since CD ETB exerts natriuretic and diuretic effects. A mouse inner medullary collecting duct cell line (IMCD3) was treated with aldosterone for 48 h followed by sarafotoxin‐6c, an ETB‐selective agonist, and extracellular signal‐related kinase 1/2 (ERK) phosphorylation assessed. S6c increased the phospho/total‐ERK ratio similarly in control and aldosterone‐treated cells (aldosterone alone increased phospho/total‐ERK). Since cultured IMCD cell lines lack ETB inhibited AVP signaling, the effect of S6c on AVP‐stimulated cAMP in acutely isolated IMCD was assessed. Rats (have much higher CD ETB expression than mice) were exposed to 3 days of a normal or low Na^+^ diet, or low Na^+^ diet + desoxycorticosterone acetate. S6c inhibited AVP‐stimulated cAMP in rat IMCD by the same degree in the high mineralocorticoid groups compared to controls. Finally, S6c‐stimulated cGMP accumulation in cultured IMCD, or S6c‐stimulated nitric oxide or cGMP in acutely isolated IMCD, was not affected by prior aldosterone exposure. These findings provide evidence that aldosterone does not modify ETB effects on ERK phosphorylation, AVP‐dependent cAMP inhibition, or NO/cGMP accumulation in the IMCD. Thus, while aldosterone can inhibit endothelial cell ETB activity to promote hypertension and injury, this response does not appear to occur in the IMCD.

## Introduction

Collecting duct (CD) endothelin‐1 (ET‐1) is an important regulator of CD Na^+^ and water reabsorption; CD‐specific knockout of ET‐1 causes renal salt retention and marked salt‐sensitive hypertension (Ahn et al. 2004). This autocrine effect of ET‐1 is mediated in part by endothelin B receptors (ETB); activation of CD ETB inhibits epithelial Na^+^ channel (ENaC) activity (Bugaj et al. 2012) and vasopressin (AVP)‐stimulated cAMP accumulation (Kohan et al. 1993); mice with CD‐specific ETB knockout have salt‐sensitive hypertension (Ge et al. 2006). Thus, CD‐derived ET‐1 autocrine activation of ETB exerts a natriuretic and diuretic effect.

The CD ET‐1/ETB system is potentially subject to regulation by multiple factors that modulate CD Na^+^ and water transport. Amongst these, aldosterone is of particular interest. Aldosterone is a key stimulator of CD ENaC activity, however the hormone also increases CD ET‐1 production (Gumz et al. 2003). Such enhancement of CD ET‐1 synthesis may serve as negative feedback for the salt‐retaining effect of aldosterone. However, previous studies raise the possibility that, despite aldosterone‐stimulation of CD ET‐1, the mineralocorticoid may actually impair CD ET‐1 signaling. Aldosterone induced sulfenyl modification of ETB by reactive oxygen species in pulmonary endothelial cells caused decreased ETB‐mediated nitric oxide (NO) formation, while antagonism of the mineralocorticoid receptor reduced aldosterone‐augmented ROS production, normalized ETB‐depending NO production in endothelial cells, and improved cardiopulmonary hemodynamics in experimental pulmonary artery hypertension (Maron et al. 2012). In addition, recent studies found that aldosterone‐mediated sulfenic acid modification of ETB facilitates renal injury in experimental ischemia/reperfusion by decreasing ETB signaling; renal injury was improved by mineralocorticoid receptor antagonism (Barrera‐Chimal et al. 2016). These findings raise the possibility that aldosterone inhibits CD ETB signaling – such a scenario could potentially augment the salt‐retaining effects of mineralocorticoids through decreased autocrine CD ET‐1/ETB system induced natriuresis (and diuresis).

Based on the above considerations, the current study was undertaken to test whether mineralocorticoid treatment reduces ETB‐mediated signaling processes in the CD.

Endothelin‐1 activation of CD ETB involves several signaling systems. The inhibitory effect of ETB activation of AVP‐stimulated cAMP accumulation is mediated by Gi (Kohan et al. 1993). In contrast, CD ETB, via Gq, activates Ca^2+^‐dependent signaling, phospholipase C, ERK/Src and NO synthase 1 – processes described to partly mediate the inhibitory effects of ET‐1 on CD ENaC activity (Cramer et al. 2001; Bugaj et al. 2008). We examine both Gi (cAMP) and Gs (extracellular signal‐related kinase 1/2 [ERK1/2] and NO) pathways involved in CD ETB actions.

## Materials and Methods

### In vivo studies

Male Sprague Dawley rats weighing ~200 g were exposed to 3 days of a normal (0.3% Na^+^, Harlan Teklad, Indianapolis, IN) or low (0.01% Na^+^, Test Diet, St. Louis, MO) salt diet, or low salt diet + the mineralocorticoid desoxycorticosterone acetate (DOCA). DOCA was administered at 7.15 mg/day subcutaneously via slow release pellets (50 mg 21‐day release pellet, 3 pellets/rat for total of 150 mg/rat, Innovative Research, Sarasota, FL). All animal studies were conducted with the approval of the University of Utah Animal Care and Use Committee in accordance with the National Institutes of Health Guide for the Care and Use of Laboratory Animals.

### Acutely isolated IMCD

Rat and mouse inner medullas were removed, minced, and incubated at 37°C in 0.1% collagenase (type IV; Worthington) and 0.1% hyaluronidase (type IV, Sigma, St. Louis, MO) in Hanks Balanced Salt Solution (HBSS) containing 15 mmol/L HEPES (pH 7.4) for about 45 min. When nearly digested, 0.01% DNase (type I, Sigma) was added for 10 min. The suspension containing predominantly single cells and individual tubules was centrifuged and then subjected to two rounds of re‐suspension in 10% bovine serum albumin in HBSS and centrifuging at 400 g for 5 min. The final pellet was washed in HBSS.

### Cell culture

The mouse IMCD cell line, mIMCD3, was used for all cell culture studies. Cells were grown to confluence in 24‐well plastic culture plates in a 5% CO_2_ incubator at 37°C; 50:50 DMEM/F‐12 supplemented with 10% fetal bovine serum, 1 mg/ml penicillin and 1 mg/ml streptomycin was used as growth medium. In all studies, cells were growth arrested for 3 h prior to study by removal of serum.

### ERK assay

Cultured IMCD3 cells were incubated with vehicle or 2.8 *μ*mol/L aldosterone for 48 h. Cells were then exposed to the ETB‐specific agonist, sarafotoxin 6c (S6c, 100 nmol/L, Tocris Bioscience, Minneapolis, MN) or vehicle in HBSS for 15 min at 37°C. Total and phosphorylated ERK1/2 were measured by enzyme immunoassay (EIA) (Abcam, Cambridge, MA). Acutely isolated IMCD from rats fed a normal salt diet were treated with S6c or vehicle as described above and assayed for total and phosphorylated ERK. Note that a commercially supplied lysate is used to generate the standard curves for ERK and phospho‐ERK, hence the absolute values of ERK and phospho‐ERK are unknown – only relative values can be reported.

### Cyclic AMP assay

Acutely isolated IMCD from rats fed a normal, low salt or low salt diet + DOCA were incubated with 1 mmol/L isobutylmethylxanthine (IBMX) (to inhibit phosphodiesterases) for 30 min, then treated with 100 nmol/L S6c or vehicle in HBSS for 15 min at 37°C, followed by exposure to 100 nmol/L arginine vasopressin (AVP) for 10 min. Total cell cAMP was then assayed by EIA (Enzo Life Sciences, Farmingdale, NY) as previously described (Stricklett et al. 2006). Total cell protein was determined by Bradford assay (Bio‐Rad, Hercules, CA). IMCD3 cells were similarly treated with S6c and AVP, followed by determination of cAMP and total cell protein as above.

### Cyclic GMP assay

Studies were done in cultured IMCD3 and acutely isolated IMCD from rats fed a normal salt diet or low salt diet + DOCA as described above. Cells were incubated with IBMX for 30 min and then treated with 100 nmol/L S6c or vehicle in HBSS for 15 min at 37°C. Total cell cGMP was then assayed by EIA (Enzo Life Sciences) and total cell protein determined. In a separate set of studies, cells were exposed to HBSS alone or containing 100 nmol/L S6c with 1 mmol/L L‐NAME added during the IBMX pre‐incubation.

### Nitric oxide assay

Studies were done in cultured IMCD3 and acutely isolated IMCD from rats fed a normal salt diet or low salt diet + DOCA as described above. Cells were incubated with 10 *μ*mol/L 4‐amino‐5‐methylamino‐2′,7′‐difluorofluorescein diacetate (DAF‐FM‐DA, Invitrogen, Waltham, MA) in HBSS for 30 min at 37°C, then washed and allowed to rest for 30 min in HBSS. Cells were then treated with 100 nmol/L S6c or vehicle in HBSS for 15 min at 37°C. Fluorescence was determined using 495 nm excitation and 515 nm emission with a SpectraMax Gemini EM microplate reader (Molecular Devices, Sunnyvale, CA). Total cell protein was also determined.

### Statistics

Data are presented as mean ± standard error. All data were analyzed by ANOVA with the post hoc Scheffe test. *P *< 0.05 was taken as significant.

## Results

### Aldosterone regulation of S6c induced ERK phosphorylation

**Figure 1:**
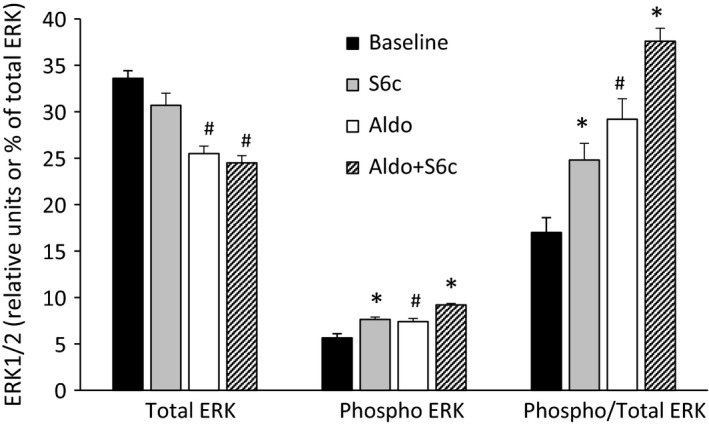
Effect of aldosterone (2.8 *μ*mol/L for 48 h) on S6c (100 nmol/L for 15 min) stimulation of total ERK and ERK phosphorylation in IMCD3 cells. *N* = 12 per data point. *P < 0.05 versus same condition without S6c; ^#^ *P* < 0.05 versus baseline.

The first series of studies were designed to test if aldosterone altered ETB signaling in the CD via Gs proteins; ERK phosphorylation was chosen for this purpose. To accomplish this, several model systems were tested. First, acutely isolated IMCD from mice and rats were tested for the ability of S6c to stimulate ERK phosphorylation. We were unable to detect any stimulatory effect of S6c at up to 100 nmol/L and for up to 15 min on ERK phosphorylation (vehicle: total ERK – 7.3 ± 0.6, phospho‐ERK – 0.66 ± 0.04, phospho/total ERK – 8.35 ± 0.6%; S6c: total ERK – 6.4 ± 0.4, phospho‐ERK – 0.49 ± 0.04, phospho/total ERK – 7.7 ± 0.3%; *N* = 8 each data point, values shown for rats [mice not shown but with similar results]). We next tested an IMCD cell line, IMCD3, which had been reported to have ETB‐mediated ERK phosphorylation (Hyndman et al. 2012). Notably, this cell line is derived from the mouse; rat CD cell lines are not generally available. As shown in Figure 1, exposure to 100 nmol/L S6c increased ERK phosphorylation. To test the effect of aldosterone, IMCD3 cells were pre‐incubated with 2.8 *μ*mol/L aldosterone for 48 h; this pre‐treatment had no effect on the ability of S6c to enhance ERK phosphorylation. Notably, aldosterone alone, as has been repeatedly shown, increased ERK phosphorylation.

### Aldosterone regulation of S6c inhibition of AVP‐stimulated cAMP production

**Figure 2:**
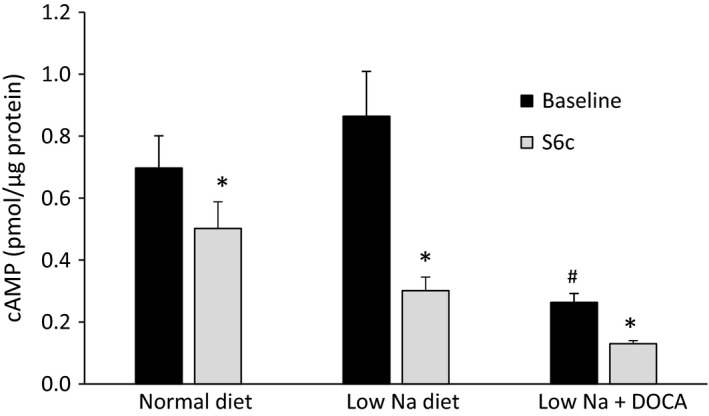
Effect of S6c (100 nmol/L for 15 min) on vasopressin (100 nmol/L for 10 min) stimulated cAMP accumulation in acutely isolated IMCD from rats given 3 days of a normal or low Na^+^ diet, or low Na^+^ diet and DOCA (7.15 mg/day subcutaneously). *N* = 12 per data point. *P < 0.05 versus same condition without S6c; ^#^ *P* < 0.05 versus normal diet baseline.

The second series of studies were designed to test if aldosterone altered ETB signaling in the CD via Gi proteins; AVP‐induced cAMP accumulation was chosen for this purpose. As for ERK signaling, several model systems were tested. Since S6c stimulated ERK phosphorylation in IMCD3 cells, this approach was attempted first. While AVP increased cAMP accumulation in these cells, S6c had no inhibitory effect on AVP‐induced cAMP (vehicle alone – no cAMP detected, AVP – 0.33 ± 0.06 pmoles cAMP/*μ*g protein, AVP+S6c – 0.29 ± 0.03 pmoles cAMP/*μ*g protein, *N* = 8 each data point). This finding is in accord with previous studies showing the Gi protein‐mediated effects of ET‐1 are frequently lost when CD cells are cultured (Woodcock and Land 1992). Consequently, acutely isolated IMCD were examined. Acutely isolated mouse IMCD do manifest ET‐1 inhibition of AVP‐stimulated cAMP accumulation (Strait et al. 2007), however the amount of tissue obtained, the magnitude of AVP‐stimulated cAMP, the degree of ETB‐mediated inhibition of AVP‐stimulated cAMP, and the amount of ET receptors on acutely isolated mouse IMCD are far less for mouse as compared to rat IMCD (unpublished observations by our laboratory). Hence, acutely isolated rat IMCD were examined. S6c substantially inhibited AVP‐stimulated cAMP content in acutely isolated rat IMCD (Fig. 2). Placing rats on a low salt diet for 3 days (to increase endogenous aldosterone) did not alter the magnitude of S6c inhibited AVP‐stimulated cAMP. Further, treatment with DOCA for 3 days at supraphysiological doses on top of administration of a low salt diet also did not affect the degree of S6c inhibition of AVP‐induced cAMP.

### Aldosterone regulation of S6c‐stimulated cGMP and NO accumulation

**Figure 3:**
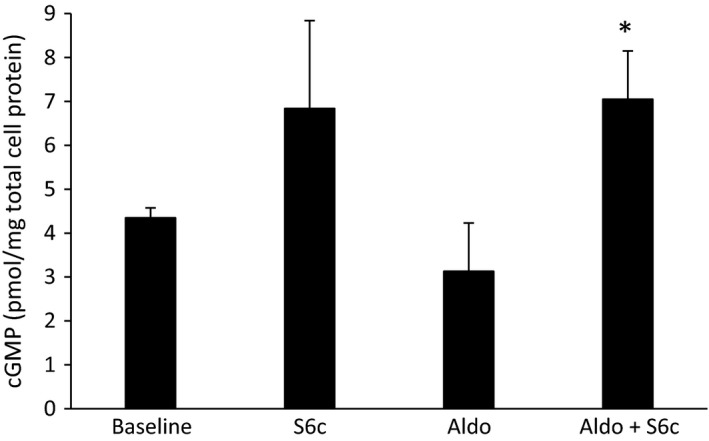
Effect of aldosterone (2.8 *μ*mol/L for 48 h) on S6c (100 nmol/L for 15 min) stimulation of cGMP in IMCD3 cells. *N* = 6 each data point. *P < 0.05 versus same condition without S6c.

**Figure 4:**
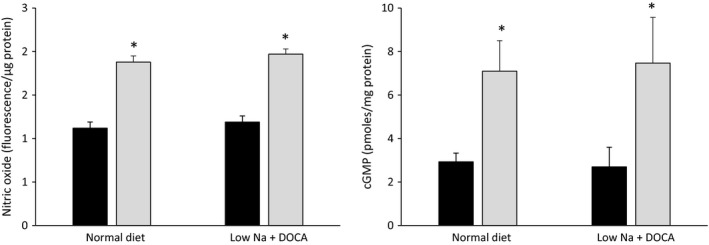
Effect of S6c (100 nmol/L for 15 min) on NO (left panel) and cGMP (right panel) in acutely isolated IMCD from rats given 3 days of a normal Na^+^ diet or low Na^+^ diet + DOCA (7.15 mg/day subcutaneously). *N* = 9–10 each data point. *P < 0.05 versus same condition without S6c.

The final set of studies tested whether aldosterone altered ETB‐stimulated NO and/or cGMP accumulation. No NO signal could be detected using DAF‐FM‐DA in IMCD3 cells. As a surrogate, S6c‐stimulated cGMP levels were assessed in IMCD3 cells: S6c comparably increased cGMP quantitatively in the presence or absence of prior exposure to 2.8 *μ*mol/L aldosterone for 48 h (Fig. 3). That S6c‐stimulated cGMP was NO‐dependent in IMCD3 cells was supported by the finding that 1 mmol/L L‐NAME prevented S6c‐stimulated cGMP accumulation (1.9 ± 0.4 pmoles cGMP/mg protein in control ± L‐NAME vs. 2.1 ± 0.3 pmoles cGMP/mg protein in S6c ± L‐NAME, *N* = 5). In acutely isolated rat IMCD, S6c increased both NO (as assessed by DAF‐FM‐DA) and cGMP levels (Fig. 4). Placing rats on a low salt diet plus DOCA for 3 days did not alter the magnitude of S6c induced NO or cGMP (Fig. 4).

## Discussion

Determination of the existence, or lack thereof, of aldosterone modification of CD ETB signaling is important since aldosterone, via modification of ET‐1 autocrine actions in the CD, could potentially impact urinary salt and water excretion and blood pressure. As discussed earlier, aldosterone stimulates CD ET‐1 production, while ET‐1, via activation of ETB in the CD, exerts natriuretic, diuretic and antihypertensive effects (potentially functioning as negative feedback for the salt‐retaining effects of aldosterone). However, aldosterone can inactivate ETB signaling, thereby potentially offsetting the stimulatory effect of aldosterone on ET‐1 production. The aldosterone‐mediated ETB inactivation has been described in the context of down‐regulation of pulmonary endothelial or renal NO production, most likely through reduced NOS3 activity (Maron et al. 2012; Barrera‐Chimal et al. 2016). However, ETB activates multiple pathways that could impact CD salt and water reabsorption, hence the current study assessed the initiating processes in such ETB signaling: activation of G‐protein‐dependent pathways.

The current study did not find evidence that aldosterone regulates CD ETB coupled Gs or Gi protein signaling. Looking at both of these signaling pathways was important in assessing ETB signaling since both have been reported to be involved in ETB actions in the CD. As stated previously, ET‐1 stimulation of Gq in CD ultimately leads to activation of phospholipase C, MAPK/Src and Ca^2+^‐dependent signaling – processes described to partly mediate the inhibitory effects of ET‐1 on CD ENaC activity (Cramer et al. 2001; Bugaj et al. 2008). Importantly, ET‐1 activation of Gq is dependent upon palmitoylation, but not phosphorylation, of ET receptors (Cramer et al. 2001). Palmitoylation of ETB has been reported to occur at cysteine residues (amino acids 402, 403 and 405) (Okamoto et al. 1998); aldosterone, via cystenic acid modification of these cysteines, can lead to inactivation of ETB signaling (Maron et al. 2012; Barrera‐Chimal et al. 2016). In particular, mutation of these 3 cysteines in ETB leads to loss of Gs signaling (Okamoto et al. 1998). Endothelin‐1, via ETB, also activates Gi signaling which inhibits AVP‐stimulated water reabsorption in the CD (Kohan et al. 1993). Mutation of ETB by deleting Cys^403^ and Cys^405^ prevented ETB coupling with Gi and abolished its inhibitory effect on adenylyl cyclase activity (Okamoto et al. 1998). Thus, key effects of CD ETB activation involve both Gs and Gi signaling; the lack of an effect of aldosterone on ETB‐mediated coupling to these pathways does not support the notion that aldosterone inactivates key CD ETB‐mediated signaling processes. Finally, we are able to compare our studies directly with those examining aldosterone inhibition of ETB‐stimulated NO formation in other cell types (Maron et al. 2012) (Barrera‐Chimal et al. 2016); the lack of an effect of aldosterone on S6c‐stimulated NO‐dependent cGMP accumulation in IMCD3 cells or on S6c‐stimulated NO and cGMP in acutely isolated rat IMCD indicates a clear difference in aldosterone regulation of ETB in endothelial as compared to IMCD cells.

In summary, the current studies suggest that aldosterone does not modify ETB signaling in the IMCD. Notably, a recent study using CD‐specific ET‐1 knockout mice is in accord with the results of the current study. In these experiments, CD‐specific ET‐1 knockout mice failed to escape from the salt‐retaining effects of mineralocorticoids – renal Na^+^ retention and blood pressure rose over the 12 days of mineralocorticoid administration (Lynch et al. 2015). If mineralocorticoids inactivated CD ETB, then one would have expected CD ET‐1 KO to have a relatively modest, if any, effect on the pattern of escape from the Na^+^‐retaining effects of mineralocorticoids. Thus, taken together, our and previous studies suggest that the IMCD ET‐1/ETB largely functions as a negative regulator of aldosterone salt‐retaining effects in the IMCD and that aldosterone does not inhibit the activity of this compensatory system. Whether this lack of IMCD ETB signaling modulation by aldosterone occurs in the outer medullary and cortical CD, as well as in other nephron segments and renal cell types, remains to be determined.

## Conflict of Interest

None.
